# Collagen Hydrolysates for Skin Protection: Oral Administration and Topical Formulation

**DOI:** 10.3390/antiox9020181

**Published:** 2020-02-22

**Authors:** Gabriel Aguirre-Cruz, Arely León-López, Verónica Cruz-Gómez, Rubén Jiménez-Alvarado, Gabriel Aguirre-Álvarez

**Affiliations:** 1 Uni-Collagen S.A. de C.V., Arnulfo González No. 203, El Paraíso, C.P. 43684 Tulancingo, Hidalgo, Mexico; aguirre98_4@hotmail.com (G.A.-C.); vero080173@hotmail.com (V.C.-G.); 2 Instituto de Ciencias Agropecuarias, Universidad Autónoma del Estado de Hidalgo, Av. Universidad Km. 1, C.P. 43600 Tulancingo, Hidalgo, Mexico; arlely@hotmail.com (A.L.-L.); ruben_jimenez@uaeh.edu.mx (R.J.-A.)

**Keywords:** peptide, skincare, extracellular matrix, hydrolyzed collagen, skin aging

## Abstract

Antioxidants are molecules that delay or inhibit the oxidation of other molecules. Its use significantly increased in recent years in the diet of people. Natural antioxidants are replacing the use of synthetic antioxidant ingredients due to their safety, nutritional, and therapeutic values. Hydrolyzed collagen (HC) is a popular ingredient considered to be an antioxidant. This low molecular weight protein has been widely utilized due to its excellent biocompatibility, easy biodegradability, and weak antigenicity. It is a safe cosmetic biomaterial with good moisturizing properties on the skin. The antioxidant properties of HC are conditioned to the size of the molecule: the lower the molecular weight of peptides, the greater the ability to donate an electron or hydrogen to stabilize radicals. The antioxidant capacity of HC is mostly due to the presence of hydrophobic amino acids in the peptide. The exact mechanism of peptides acting as antioxidants is not clearly known but some aromatic amino acids and histidine are reported to play an important role in the antioxidant activity. Oral ingestion of HC increases the levels of collagen-derived peptides in the blood torrent and improves the skin properties such as elasticity, skin moisture, and transepidermal water loss. Additionally, daily intakes of HC protect the skin against UV melasma, enhances the fibroblast production and extracellular matrix of the skin. HC has been identified as a safe cosmetic ingredient for topical formulations with good moisturizing properties at the stratum corneum layer of the skin. It reduces the effects of skin aging (dryness, laxity, and wrinkles). The use of HC as a principal ingredient in safe formulations for skin protection was reviewed and compared when it is used by topical and/or oral administration.

## 1. Introduction

The skin is an important organ of the human body and acts as a barrier against external environmental factors such as sunlight exposition, temperature, dust, bacterial attack, etc. Skin aging is a natural and unavoidable process that involves oxidative activity during the metabolism of body tissues. The oxidative process results in the generation of free radicals with one or more unpaired electrons in a reactive state. The skin possesses its own antioxidant defense against this oxidation process at subcellular compartments, organelles, as well as the extracellular space [1]. 

Aging of the skin has been widely studied in order to understand its molecular mechanisms [2] as well as factors that affect this condition. It is well-known that skin aging is the result of the combination of both intrinsic and extrinsic effects [3]. Intrinsic aging involves inherent factors related to age [4], in a neuroendocrine aspect [5], while extrinsic aging is the result of external factors such as ultraviolet A (UVA) and ultraviolet B (UVB) radiation exposure [6,7,8,9], environmental pollution [10,11], and production of ROS [12]. 

In recent years the use of antioxidants as a functional ingredient in the diet of people increased significantly. A popular ingredient considered to be an antioxidant is collagen in its hydrolyzed form. Collagen is a fibrous protein highly demanded in biomedical and cosmetic industries due to its benefits on the skin, biocompatibility, bioactivity, and weak immunogenicity [13]. This biomaterial is composed of repetitive triplets of amino acids glycine (Gly), proline (Pro), and hydroxyproline (Hyp). Collagen can be isolated from several sources resulting in a difference in its chemical and thermal properties [14]. Proteins in their native form such as collagen present low solubility in water due to its high molecular weight of approximately 300 kDa [15]. These natural properties present difficulties in their application to cosmetics and edible products. However, the degradation of collagen produces hydrolyzed collagen (HC), which consists of segmented proteins with low molecular weight (Mw) between 1 kDa and 10 kDa [16]. HC is obtained by denaturation of native collagen followed by an enzymatic process that breaks down the protein chains into small peptides cleaving the protein at specific amide bonds [17,18,19]. The use of natural peptides like collagen hydrolysates has been widely utilized due to their excellent biocompatibility, easy biodegradability, and weak antigenicity [20]. Some protein hydrolysates from casein, wheat gluten, lactalbumin, and soya sources have been studied due to their potential anti-aging properties and fibroblast renewal in monolayered cell culture [21]. However, less is known about the benefits of HC on the skin and its properties as an anti-aging, UV protector, enhancer of fibroblast production and extracellular matrix (ECM), moisturizer, and nutraceutical. This review covers the current understanding of HC applied by topical and/or oral administration as a principal bioactive component for skin protection. 

## 2. Topical Formulation

Topical formulations are commonly applied directly to the skin with the help of a vehicle or base for a specific site of the body. Formulations are designed to maximize the penetration of an active or functional ingredient into the skin. The relationship between nutrition and skin has been a topic of interest for scientists in the field of cosmetic and pharmaceutical areas [22]. From these roots came the concept of “cosmeceutical”, which is the blend of the terms cosmetic and pharmaceutical, and refers to something between a drug and a cosmetic [23].

### 2.1. Role of the Skin in Protection

The skin provides a barrier between the body and its environment. It allows the control and functionality of other corporal factors. It helps to maintain a constant body temperature. This function is regulated by the hypothalamus. Lipids and ceramides of the external layers of the skin provide a barrier against the excessive loss of water [24]. Dryness of the skin could appear if these emerged lipids are removed by the use of detergents. The skin gives protection against the effect of sunlight exposure. Melanin is the natural pigment in the skin that absorbs ultraviolet (UV) light [25]. The skin protects against chemical and microbiological attacks [24]. Lamellar granules release intercellular lipids in the underlying stratum granulosum. These lipids produce a lipid barrier composed for ceramides (50%), free fatty acids (15%), and cholesterol (25%) [26,27]. Its pH varies from 4.5 to 6.5 depending on the body location evaluated. Severe changes to the lipid barrier pH could lead to undesirable bacteriological invasion, sensitiveness, and diverse forms of skin dermatitis. Regarding the resistance to mechanical tension, the skin has sensory elements that identify sensations of pressing on its different forms (shape, weight, surface, deep, stinging, touch, etc.), temperature, and mechanical traction. These characteristics help to interact and react with the environment through sensory nerve fibers present in the dermis and connected to the rest of the nervous system [28].

### 2.2. Routes of Peptide Penetration onto the Skin

**Figure 1:**
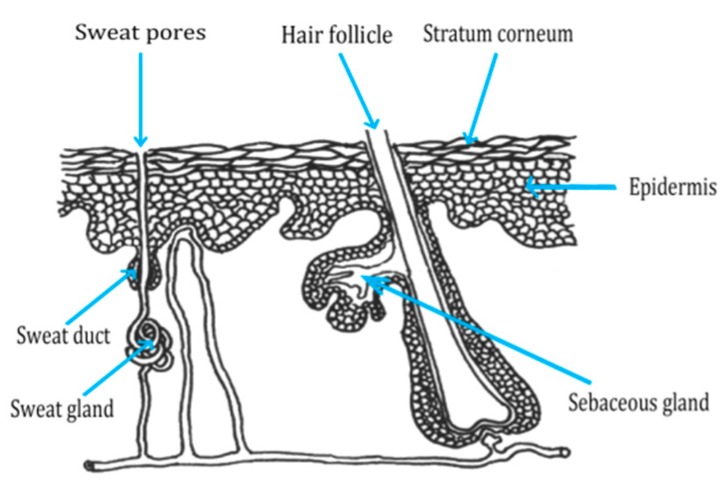
Schematic representation of three pathways for peptide penetration onto the skin. Adapted from [30].

Drug molecules, as well as collagen peptides, can penetrate the skin through three potential pathways (Figure 1). The first way is through the sweat ducts, the second is through the hair follicles and sebaceous glands, or the third way consists of penetration directly across the stratum corneum (SC) [29].

**Figure 2:**
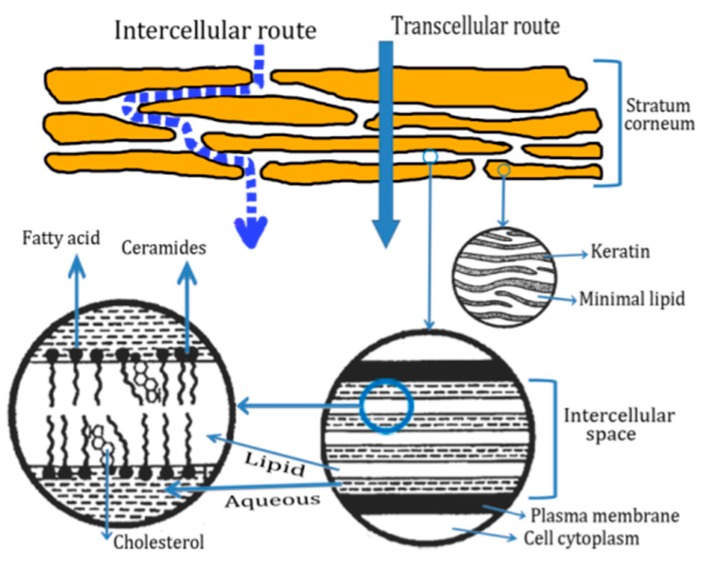
Schematic representation of stratum corneum and two micro routes for peptide penetration. Adapted from [30].

The SC of the skin represents the main barrier to the penetration of cosmetic products. The penetration of active ingredients in the skin can be carried out by two routes as illustrated in Figure 2. The intercellular path is the most common route for penetration of drugs and cosmetic products in the skin. SC is composed of multiple lipid bilayers of ceramides, fatty acids, and cholesterol. The corneocytes are embedded in a multi-lamellar lipid matrix in a fashion often described as "brick and mortar structure [31]. For the transcellular route, different pathways of circumventing the SC have been developed by the selection of proper drugs, the addition of liposomes, previous hydration of the SC, and chemical penetration enhancers. Other aggressive ways where SC is bypassed include: microneedle array, stratum corneum ablated, follicular delivery, electrically assisted methods (ultrasound, iontophoresis, electroporation, etc.) [29,30]. 

### 2.3. Transdermal Penetration of HC on the Skin

Topical cosmetic formulations are prepared with natural moisturizers from vegetal or animal sources. However, HC has been identified as a cosmetic ingredient with good moisturizing properties at the SC layer of the skin. Peptides are ingredients extensively used in the preparation of cosmetic products for structural protection of the skin, to reduce the effects of skin aging, and other treatments oriented to the beauty and health of the skin [32]. Since 1985, HC has been considered a safe ingredient for use in cosmetic formulations [33]. The Cosmetic Ingredient Review experts panel reaffirmed this consideration in a review published in 2006 [34]. According to the report related to the safety assessment of skin and connective tissue-derived proteins and peptides as used in cosmetics, the voluntary cosmetic registration program data showed that ingredients with the greatest number of uses are HC with 543 formulations; the majority of uses as a live-on skincare product. This report also states that HC is practically non-toxic when administered dermally. Subchronic dermal studies resulted in negative for systematic toxicity [35]. However, one of the main problems for topical applications is their low permeability to penetrate the skin. The larger the peptide (beyond six or seven amino acids), the less likely it is to reach the deeper layers [36]. It is well-known that permeation of the outer layers of the skin depends on different factors such as: physicochemical properties of the substance (molecular size, stability, binding affinity, solubility); the time-scale of permeation; thickness and components of the skin; cutaneous metabolism; site, area, and duration of application and properties of the transdermal device [37,38]. Some parameters have been suggested to enhance and secure the delivery of topical products on the skin. These parameters were related to molecular weight below 500 Da, partition coefficient octanol/water between 1 and 3, melting point below 200 °C, aqueous solubility > 1 mg mL^–1^, and few polar centers [30,38]. 

Zhang and co-workers conducted an experimental setup to assess the transdermal permeation effect of HC from deer sinew on mouse skin as well as the skincare protection of percutaneous proteins [39,40]. They found that approximately 8.0% of HC with a molecular weight between 5 and 13 kDa could penetrate the skin. HC also promoted cell proliferation and collagen I secretion in fibroblast cells. These results suggested that HC may be used as a cosmetic biomaterial for topical applications to protect the skin against oxidative stress and premature skin aging. It is important to make a distinction between HC and native collagen. The native state of collagen is less soluble in water and showed better physiological effects compared to HC and gelatin when keratinocyte culture experiments were carried out in vitro [40,41]. Native collagen is not able to penetrate the SC of the skin due to its high Mw calculated around 300 kDa [15].

Another experiment was carried out in an animal model experiment oriented to the evaluation of the transdermal efficiency of fish scales collagen peptides (FSCPs) [42]. The hydrolysates were fractioned into four different groups with an Mw of 4.5, 3.5, 2.0, and 1.3 kDa. After six weeks of topical application with nonwoven fabric containing 25 μg FSCPs, the SC layer was penetrated considerably for FSCPs. According to the Franz type diffusion cell model, the best transdermal penetration of FSCPs was observed with 4.5 and 3.5 kDa samples in the first four hours. Both treatments also proved to have the highest penetration depth due to their coil-like structure in the epidermis. These results were associated in the same experiment with a formulated FSCP-based skin essence mask prepared with three different concentrations of FSCPs (5%, 7%, and 10%) and applied to 30 Taiwanese women. After 30 days of topical application in the facial skin, the 10% concentration reported the higher increments in skin moisture content and relatively elasticity suggesting that FSCPs penetrated efficiently the SC. 

### 2.4. Anti-Aging Benefits

The use of topical formulations based on peptides to prevent the development of cutaneous aging is ideal for cosmetic preparations due to their active ingredients and bio-mimetic molecules with biological activity [43]. The skin aging process produces fine lines and wrinkles in facial parts associated with expression movements such as the periocular and perioral areas. As a result, it can be challenging for scientists, physicians, and patients to develop topical treatments oriented to address the challenge of treating these expressive facial areas [44].

Swuatschek and colleagues isolated and characterized collagen from marine sponge *Chondrosia reniformis* Nardo for the preparation of two different collagen-based formulations and evaluation of their effects on biophysical skin parameters [45]. Eleven female and six male volunteers were applied with 75 mg of each preparation in 25 cm^2^ area of the volar forearm for a period of 12 h. The physiological skin pH between 4.8 and 5.5 was measured after 12 h of application. This pH protects the skin from microorganisms. Regarding skin moisturizing at the SC, a slight increase in hydration was observed between the collagen-based products and control. Skin surface lipids increased between 140 and 180 μg after 1 h of application.

### 2.5. Creams and Lotions with Mosturizing Action

Sensitive skin is a clinical condition between people that suffer dry skin problems, impaired barrier function, and a tendency to erythema and desquamation. Berardesca and co-workers developed a moisturizing lotion with hydrolyzed collagen to be applied in the morning and evening in 40 female subjects affected by non-lesional atopic sensitive skin [46]. After four weeks of topical applications, there were reported significant improvements in hydration, skin surface smoothness at the microtextural level, and luminosity. The barrier function efficacy as expressed by transepidermal water loss (TEWL) was also improved. They concluded that the modulation of bacterial proliferation along the before described improvements could alleviate the symptoms of sensitive skin due to the benefit of cosmeceutical proteins such as HC [47]. Another study oriented to immediate and long-term clinical benefits of topical application of hydrolyzed collagen-based formulation was carried out to evaluate HC efficacy on periocular and perioral wrinkles [44]. Topical line treatments reduced wrinkles significantly within minutes of the initial application. After three months of treatment, continuous improvements were attributed to the effect of peptides, which have been shown to prevent MMP-induced damage to the extracellular matrix (ECM) [48].

Collagen products extracted from marine sources like alga and fish exhibit properties related to the retention of water at the superficial part of the SC. The effects of skin hydration and firmness from cosmetic formulations containing compounds derived from algae and fish collagen were conducted in medium-term (two weeks) and short-term (20 min) trials [49]. Higher values of hydration were obtained with creams enriched with the marine compounds compared to the creams without collagen. These results suggested that marine compounds brought an additional moisturizing potential. For short-term experiments, the 10% serum exhibited the most prominent firming effect. 

### 2.6. Dry Skin as a Trigger for Skin Aging

Dry skin problem has been associated with the SC of the epidermis. Therefore, big efforts have been carried out by researchers and cosmetologists in order to handle this issue by developing new formulations based on humectants (glycerin, propylene, urea, and sodium lactate), occlusive moisturizers (petrolatum, mineral oil, and paraffin) and emollients (lipids and oils) [50,51,52]. It is commonly thought that a moisturizer adds water to the skin, however, this is misinterpreted because it works by preventing or reducing water evaporation from the skin. This action promotes hydration of the skin from within [53]. Keratinization process gives origin to the formation of a semipermeable membrane commonly called the lipid barrier, which reduces the loss of water in the skin. This hydro-lipid film is a vital component for maintaining the skin barrier and level of acidity. Individual skin pH depends on many factors such as endogenous skin moisture, the composition of apocrine and eccrine sweat. Changes in skin pH above 6.5–8 act as an irritant on the protective barrier because of the changes in the skin microbial flora and the activity of enzymes at the upper layers of the epidermis. Ciszek [54] studied a group of 49 women to compare the effect of topical collagen gels on the skin and to demonstrate whether topical applications change the pH of the skin, particularly at their forehead and left cheek areas. After 20 and 60 min of topical applications, the results indicated that all collagen gel treatments produced a significant change in skin pH compared to the base line. The mean physiological pH value of the forehead (5.67) was slightly lower than the left cheek (5.76) area. Amino acids of the old cells in SC give a deposit of water that maintains the flexibility and water content of this layer due to their hygroscopic properties [55]. This deposit of amino acids accompanied by other substances is known as the natural moisturizing factor (NMF) of the skin. NMF is a complex mixture of water-soluble compounds within the corneocytes [56]. This NMF varies depending on both carboxylic acids and 2-pyrrolidine content. These are natural components in the skin and effective humectants that help to maintain the moisture balance of the skin [57]. Water flows in the direction from the dermis to epidermis. Nevertheless, some water is lost due to evaporative diffusion. TEWL provokes a gradient of humidity concentration, resulting in a water content higher at the dermis level and decreasing at the surface of the skin [58]. The SC is a very effective barrier against loss or gain of moisture [24]. The water content at the SC level is not uniformly distributed through its thickness, the more superficial layers are in equilibrium with the ambient water content, while the deeper layers exchange their free water with the epidermis [59]. 

### 2.7. Hydration of the Skin

Water is a vital element for skin and hydration becomes fundamental for its health and beauty. Commonly, topical applications of cosmetic products are oriented to the maintenance and improvement of hydration in the skin [60,61]. The hydration state of skin reaches an equilibrium due to diffusion of water due to relative humidity, retention of water for ceramides and lipids, loss of water by evaporation and capacity of the epidermis to retain water in its structure (loss of flexibility, wrinkles, and excess of a dry and scaly skin surface). These variations of the aqueous content in the skin determine the state of hydration, which is the first criterion to establish the cutaneous typologies of the skin (hydrated or dehydrated skin). Water in dermis and epidermis represents 20% of the total water content in the body [62]. A hydrated and healthy skin reaches about 70% water content at the dermis layer and 30% at the outer layer (stratum corneum) [63,64]. Water in biological systems like the skin is hydrogen-bonded either to proteins (collagen) or intermolecularly to other water molecules. Water that is not bound to proteins is commonly called tetrahedron or free water [62,63,64,65]. In young skin, most of the water is bound to collagen resulting in great importance for the structural and mechanical properties of proteins and their mutual interactions. However, in aged skin, water is found in the tetrahedron form. Thus, the more hydrophobic and folded the protein is, the less interaction with water will occur; therefore, water binds to itself instead [66]. Water in the skin goes from dermis to epidermis and is dissipated to the exterior. However, retained water is located between lamellar granules of the skin as well as the inner part of the corneocytes. The static hydration of the SC is utilized to increase both the plasticity of the epidermis and the hydrophilic properties of keratin [31]. Several modern techniques based on imaging and spectroscopy have been reviewed for hydration assessment of the skin [67,68,69]. Although traditional methods remain in use so far, they can be supported with emerging technologies for a more complete assessment of the skin hydration levels [70,71,72,73,74]. 

## 3. Role of Hydrolyzed Collagen as an Antioxidant Ingredient

Antioxidants are molecules with the capacity to metabolize free radicals and consequently neutralize the deleterious effects induced by oxidative stress [75]. They are classified as primary or secondary antioxidants according to their antioxidant mechanisms [76]. The primary antioxidants or interceptive, the so-called chain-breaking antioxidants, are free radical acceptors that delay or interrupt the propagation steps of oxidation. In the course of the neutralization process, the antioxidant loses a proton and is transformed into a radical. Secondary or preventive antioxidants act through several mechanisms. These antioxidants have the ability to slow down the rate of oxidation, but they are not able to convert reactive species to more stable products. This type of antioxidant can chelate pro-oxidants metals and deactivate them, replenish hydrogen to primary antioxidants, deactivate single oxygen, act as scavengers, or absorb UV radiation [77]. According to Esfandi et al. [78], there are two main mechanisms by which antioxidant molecules can deactivate free radicals: hydrogen atom transfer (HAT) and single electron transfer (SET). Both of them may occur in parallel, but one can dominate depending on the structure of the antioxidant peptide. Tyrosine containing peptides can act mainly through the HAT mechanism while cysteine and histidine peptides act mainly via the SET mechanism.

Uncontrolled production of free radicals that attack macromolecules such as membrane lipids, proteins, and DNA may lead to many health disorders such as cancer, diabetes mellitus, neurodegenerative, and inflammatory diseases with severe tissue injuries [79]. Additionally, deterioration of foods has been identified due to oxidation of lipids and the formation of undesirable secondary lipid peroxidation products. Traditionally, the use of synthetic antioxidants such as butylated hydroxytoluene (BHT), butylated hydroxyanisole (BHA), tertbutylhydroquinone (TBHQ), and propyl gallate (PG) have been used to retard peroxidation processes along with their strict control due to potential health issues [80]. Hence, the use of natural antioxidants is replacing the use of these synthetic antioxidant ingredients due to their safety, nutritional, and therapeutic values.

León-López and co-workers demonstrated that hydrolyzed collagen (HC) from sheepskins is a natural antioxidant that showed good antioxidant activity as evaluated by 2,2-diphenyl-1-picrylhydrazyl (DPPH) and 2,2′-azino-bis (3-ethylbenzothiazoline-6-sulphonic acid) (ABTS) techniques. They found that the antioxidant properties of HC depended on the size of the molecules; the lower the molecular weight of peptides, the greater the ability to donate an electron or hydrogen to stabilize radicals. The best treatment was observed when HC was obtained after four hours of enzymatic hydrolysis with an average molecular weight of 5 kDa [81]. 

Recently, the study of peptides derived from marine protein hydrolysates has confirmed potential antioxidant properties. These studies considered different marine sources such as jumbo squid, oyster, blue mussel, hoki, tuna, cod, capelin, scad, mackerel, Alaska pollack, yellowfin sole, and yellow stripe trevally [82,83,84,85,86,87,88,89,90,91,92,93]. Some of the benefits of these marine bioactive ingredients consisted of their ability to scavenge free radicals. Also, preventing oxidative damage by interrupting the radical chain reaction of lipid peroxidation [83,84]. Mendis et al. [82] showed that the antioxidant potency of HC is mostly due to the presence of hydrophobic amino acids in the peptide. These results indicated that peptides derived from marine fish proteins have greater antioxidant properties in different oxidative systems. However, the exact mechanism of peptides acting as antioxidants is not clearly known but some aromatic amino acids and histidine are reported to play an important role in the activity [94]. The valuation of the antioxidant capacity of natural products may involve a sequential multifaceted approach including a Folin reagent; an oxygen radical absorbance capacity (ORAC) assay; metal-reducing activity, like ferric reducing antioxidant power (FRAP); the inhibition of low-density lipoprotein (LDL) peroxidation; the inhibition of tyrosine nitration; and a cellular antioxidant activity (CAA) assay. The antioxidant capacity of a natural product will essentially depend on the bioavailability of the specific mixture of compounds of the product, their synergistic interactions to yield the final antioxidant response at the cellular level [75]. 

## 4. Oral Administration of HC

### 4.1. Absorption of HC into the Blood Torrent

Beauty comes from the inside and that means that nutrition is a key point for healthy skin and therefore decelerating the skin aging process [95,96]. Ingestible food products that are formulated for beauty purposes are described now as “nutricosmetics” [97]. 

Several works have demonstrated that the levels of collagen-derived peptides in the blood torrent increased significantly after HC oral ingestion, suggesting that collagen molecules are absorbed into human plasma [98,99]. Ohara et al. [100] formulated a study in five healthy males looking at a comparison in quantity and structure of hydroxyproline-containing peptides in human blood after oral ingestion of gelatin hydrolysates from fish and skin sources. They concluded that 30% of all detected hydroxyproline (Hyp) corresponded to Hyp-containing peptides compared to the free form of Hyp. Yazaky and co-workers [101] also analyzed the plasma concentrations of collagen-derived peptides into the bloodstream and skin in twelve healthy individuals who ingested (300 mg/Kg body weight) either high tri-peptide containing collagen hydrolysates (HTC-Col) or ow tri-peptide containing collagen hydrolysates (LTC-Col). They concluded that HTC-Co showed peak richness in tri-peptide components in the blood after oral ingestion (13.6%). 

In 2019, Skov et al. [102] carried out a clinical study to investigate the postprandial (after eating a meal) absorption of collagen from beef bone and elucidated the impact of exogenous enzymatic hydrolysis on absorption rate and bioavailability. Ten healthy male subjects ingested either 35 g enzymatically hydrolyzed collagen protein (EHC), non-enzymatically hydrolyzed collagen (NC), or placebo (250 mL water) during three consecutive days. The study concluded that the absorption rate and bioavailability of three particular amino acids (Gly, Pro, and Hyp) were significantly higher after oral administration of EHC. The results gathered suggested that ingestion of collagen in its hydrolyzed form promotes a higher absorption rate in the postprandial plasma concentration of total amino acids compared to the NC and placebo group. 

Human consumption of dietary supplements such as hydrolyzed collagen has been deemed to be safe. Lopez-Morales et al. [103] conducted a detailed experimental work to characterize HC with different techniques such as ultraviolet detection (SEC–UV), reverse phase chromatography coupled to electrospray ionization ion mobility quadrupole time-of-flight spectrometer (RP–HPLCESI–IMS–QTOF), and shaped-pulse water-suppression one (1D)- and two-dimensional (2D) proton nuclear magnetic resonance spectra. Safety and toxicity of HC were assessed in vitro by using CaCo-2 and HepG2 cell lines. They found that HC is safe and no toxic on the evaluated cell lines. The mass distribution pattern obtained by SEC resulted in a range from 1.35 kDa to 17 kDa and RP–HPLCESI–IMS–QTOF showed a range from 2 kDa to 14 kDa. These low molecular weight peptides were soluble in water and able to be digested, absorbed, and transported to the systemic circulation system as peptides in the small intestine [104]. The absorption of HC tripeptides (Gly-Pro-Hyp) or dipeptides (Pro-Hyp) in the bloodstream was demonstrated in rats’ model by Yamamoto and co-workers. This occurs as early as 10 min after oral administration [105]. 

### 4.2. Improvement of Skin Properties

In 2017, Genovese et al. [106] performed a clinical study of 120 healthy volunteers for 90 days. They were instructed to ingest either 50 mL of nutricosmetic formulation (hydrolyzed collagen, hyaluronic acid and N-acetylglucosamine, borage oil, and other ingredients such as vitamins, minerals antioxidants, and additional bioactive ingredients) or 50 mL of placebo (water and other ingredients such as flavors, organic acids, and soybean polysaccharide). The histological analysis revealed that oral supplementation of the nutricosmetic formulation produced an improvement in the structure and stratification of the epidermal layers. The collagen fibers’ structural architecture within the dermis was improved. A complementary part of this study was the self-assessment questionnaire. The most relevant answers commented that 95% of the subjects agreed their skin was more hydrated. They answered their skin was more elastic (91.6%), stronger (81.7%), and thicker (91.7%). 

Another clinical study was conducted in 85 Chinese female subjects treated to low and high free-formed ratios of Pro-Hyp and Hyp-Gly derived from fish source [107]. Five-gram samples were ingested for eight weeks. Three physiological measurements were evaluated on the skin; the skin moisture showed a significant increase compared to the placebo. In regard to skin elasticity, a significant improvement of facial skin elasticity after oral ingestion of collagen hydrolysates resulted. The higher ratios of Pro-Hyp and Hyp-Gly showed the best results in skin elasticity. This treatment also appeared to have the best results for surface skin measurements because of a significant reduction in the number of wrinkles, wrinkle area, wrinkle depth, and roughness. Other works from these authors also discovered that oral ingestion of HC decreased the area of UV spots on the skin after four weeks of oral ingestion [108]. 

Similar results on the improvement of the skin were obtained by Ito et al. [109] in 40 healthy women by the ingestion of 30-mL of fish-derived collagen and ornithine (CPO) drink. After eight weeks of supplementation, skin elasticity increased in CPO treatments. Skin moisture and TEWL were significantly attenuated on CPO groups compared with placebo. In addition, the number of skin pores was reduced in CPO treatment.

Improvements on skin properties were reported previously [110] in a clinical study carried out with fish HC administered to 25 Japanese female subjects diagnosed with dry and rough skin. The results showed that the moisture content of the SC of face-cheek, forearm, and the back of the neck increased significantly after six weeks of daily intake of 7 g of HC. Also, there were significant enhancements in skin elasticity by reducing wrinkles and lowering skin roughness.

Skin aging produces physiological changes at the dermis level and is manifested by visible signs such as dryness, laxity, and wrinkles and photoaging in the face. Schwartz and Park [111] developed a study to treat these effects of skin aging. A 1 g mixture of hydrolyzed collagen type II from chicken sternal articular cartilage, hyaluronic acid, and chondroitin sulfate was administered to 26 healthy females for twelve weeks. The study showed that oral supplementation led to a significant decrease in facial lines and wrinkles including skin dryness and scaling. 

The benefits of ortho-silicic acid stabilized in hydrolyzed marine collagen as a treatment against the skin aging process were evaluated in another study [112]. Twenty-two female and male volunteers were counseled to take, before breakfast, one capsule daily with ortho-silicic acid and hydrolyzed marine collagen. After 90 days of treatment, the authors concluded that degenerative changes of the ECM were recovered, stimulation for the synthesis of collagen type I, improvements in skin firmness, texture, and hydration were achieved. 

### 4.3. Protection of the Skin Against UV and Melasma

Ultraviolet is divided into three regions according to wavelength: UV-A (400–315 nm), UV-B (315–280 nm), and UV-C (<280). Continuous exposition to UV-B results in an aged skin with wrinkle formation [113]. The benefits of a novel oral supplement (CP) prepared with fish skin HC combined with soy peptides and aqueous extract of *Flos Chrysanthemi Alba* were investigated in a clinical study to evaluate the safety and efficacy of CP to treat melasma [114]. Sixty-two healthy female volunteers who were diagnosed with melasma by a dermatologist were included in the study. They were instructed to take 10 g of CP per day along with breakfast for a period of 60 days. The authors reported a reduction in hyperpigmentation with lighter facial skin tone after oral supplementation of CP. The melanin contrast index in the lesion area was reduced in comparison with the placebo group. Their results suggested that CP inhibited UV-B-induced pigmentation because of the antioxidant activity and tyrosinase inhibitory effects of CP. Lee et al. conducted research to study the effects of oral administration of fish scale collagen peptides on the skin protection when exposed to UV-B. They concluded that consumption of dipeptides in the form of Gly-Pro and Pro-Hyp attenuated the UV-B induced wrinkle formation. The enhancement of other skin properties was reported such as skin hydration, transepidermal water loss, and epidermis thickness [115]. 

### 4.4. Enhancement of Fibroblast Production and Extracellular Matrix of the Skin

The extracellular matrix (ECM) is made up of several types of proteins such as collagen type I, elastin, and proteoglycans, which are largely produced and secreted for fibroblasts [116,117]. Some of the changes from the aging of the skin are the structural modifications of the reticular dermis induced by loss of internal cohesion as well as the rupture and decrease of collagen and elastin fibers. This happens because there is reduced production of fibroblasts responsible for the production of collagen, and collagen fibers are fragmented and thinned [118]. However, daily intakes of food-derived collagen peptides enhance the growth of fibroblasts. Pro-Hyp and Hyp-Gly dipeptides play an important role in the proliferation of fibroblasts [119]. It has been demonstrated that the rest of the fragmented collagen inhibits synthesis of procollagen by fibroblasts, blocks their proliferation, induces a senescent fibroblastic morphology, and stimulates the expression of matrix metalloproteinases (MMP) [120]. These defects impair the structural and mechanical integrity of the dermis and, therefore, alter its functions; reduction of glycosaminoglycans content and consequent loss of water retention in the dermis due to the reduction of hygroscopic capacity of the skin. All these morphological and physiological changes are manifested in the skin as flaccidity and loss of texture.

In 2014, Proksch et al. [121] carried out a clinical study in 114 healthy female subjects to investigate the effect of the oral ingestion of 2.5 g porcine type I collagen peptides with an average Mw of 2 kDa for a period of time of eight weeks. The eye wrinkle volume parameter showed good results after four weeks of treatment. However, after eight weeks, the reduction of wrinkles reached up to 20% on average compared to the placebo. Production of procollagen type I and elastin were increased by up to 65% and 18%, respectively, after eight weeks of treatment. Fibril content increased by up to 6% compared to the placebo. It is well-known that elastic fibers are essential ECM molecules composing an elastin core surrounding by a mantle of fibrillin-rich microfibrils [122]. Previous studies have demonstrated that daily intake of collagen peptides decreases the expression levels of matrix metalloproteinase, which is responsible for collagen breakdown [123,124,125].

Borumand and Sibilla conducted research oriented to investigating the effect of a supplement composed of collagen hydrolysates from tilapia and pangasius fish, hyaluronic acid, vitamins, and minerals on skin properties [126]. The study was carried out in 294 volunteers aged 18–74 years old recruited by 40 dermatologists across five different countries. They were instructed to drink 50 mL of their food supplement once a day. The study was structured into three sections. The first section reported that 69% had either visible or significant improvement in their facial lines after 60 days of treatment. The volunteers improved their facial photoaging problems and skin dryness. The second section reported an increment in dermal collagen density. After 12 weeks of treatment, the magnitude of collagen density was greater on the face than on the forearm. The third section showed that the skin firmness increased by up to 94% when the treatment reached 130 days.

Aserrin and co-workers completed a study to assess the effect of daily oral supplementation with collagen peptides on skin hydration, collagen density, and collagen fragmentation. Human skin explants were used to study the ECM components [127]. Thirty-three female volunteers, 40–59 years old, were recruited to ingest 10 g of either fish collagen, porcine collagen, or placebo. Porcine collagen showed the best hydration increasing by up to 28% after eight consecutive weeks of intake. Results from the high-frequency ultrasound demonstrated that oral intake of collagen peptides increased the dermal echogenicity significantly immediately after four weeks of treatment. Additionally, this effect persisted after twelve weeks of treatment when compared with the baseline value. Collagen peptides significantly reduced fragmentation after supplementation. Fibers in the skin appear to be larger collagen fragments compared to the placebo and baseline values. The general morphology, glycosaminoglycans, and collagen content of the human skin explants reported that the glycosaminoglycan level in the basal epidermis increased significantly. In correlation with these results, the collagen content of the papillary dermis increased in response to the incubation with collagen peptides. However, the general morphology of the skin was not affected significantly. 

## 5. Combination of Both Topical and Oral Intake

Innovative studies have been carried out with the combination of topical application of peptides extracted from rice and oral supplementation of HC mixed with vitamin A, C, D, and Zinc. This study was developed in 60 healthy female subjects by applying biophysical and skin imaging techniques [128]. The levels of hydration at deeper layers of the epidermis were improved after one month by the topic application of cosmetic formulation with peptides. However, after 90 days, the skin elasticity and viscoelasticity increased significantly. Oral supplementation of HC improved elasticity on the forehead and nasolabial regions accompanied by the reduction of wrinkles and a decrease in skin pores on the molar region. 

## 6. Conclusions

Hydrolyzed collagen is a protein that can be easily absorbed into human plasma. It is safe and highly demanded in the nutraceutical industry due to its benefits on the skin and biocompatibility. There were plenty of reports providing evidence that oral ingestion of collagen hydrolysates promotes the growth of fibroblasts and stimulates the production of new collagen type I in the dermis. It makes the skin smoother, softer, and provides enhanced textural properties. Daily intakes of HC decrease the expression levels of matrix metalloproteinase, which is responsible for collagen breakdown. The antioxidant properties of HC are mostly due to the presence of hydrophobic amino acids in the peptide. However, more research is needed in order to investigate the exact mechanism of peptides when acting as antioxidants and its relationship with its aromatic amino acids and histidine components. HC seems to be an effective bioactive ingredient to improve dermal health and slow down the effects of skin aging.

## Abbreviations

UVA	Ultraviolet A
UVB	Ultraviolet B
SC	Stratum Corneum
ECM	Extracellular Matrix
NMF	Natural Moisturizing Factor
TEWL	Transepidermal Water Loss
MMP	Matrix Metalloproteinases
kDa	KiloDalton
HC	Hydrolyzed Collagen
